# Cardiac Energy Metabolism and Oxidative Stress Biomarkers in Diabetic Rat Treated with Resveratrol

**DOI:** 10.1371/journal.pone.0102775

**Published:** 2014-07-22

**Authors:** Klinsmann Carolo dos Santos, Camila Pereira Braga, Pedro Octavio Barbanera, Fábio Rodrigues Ferreira Seiva, Ary Fernandes Junior, Ana Angélica Henrique Fernandes

**Affiliations:** 1 Department of Chemistry and Biochemistry, Institute of Bioscience, São Paulo State University (UNESP), Botucatu/São Paulo, Brazil; 2 Institute of Biology, North of Parana State University (UENP), Bandeirantes/Paraná, Brazil; 3 Department of Microbiology and Immunology, Institute of Bioscience, São Paulo State University (UNESP), Botucatu/São Paulo, Brazil; University-Hospital of Parma, Italy

## Abstract

Resveratrol (RSV), polyphenol from grape, was studied to evaluate its effects on calorimetric parameters, energy metabolism, and antioxidants in the myocardium of diabetic rats. The animals were randomly divided into four groups (n = 8): C (control group): normal rats; C-RSV: normal rats receiving RSV; DM: diabetic rats; and DM-RSV: diabetics rats receiving RSV. Type 1 diabetes mellitus was induced with administration of streptozotocin (STZ; 60 mg^−1^ body weight, single dose, i.p.). After 48 hours of STZ administration, the animals received RSV (1.0 mg/kg/day) for gavage for 30 days. Food, water, and energy intake were higher in the DM group, while administration of RSV caused decreases (p<0.05) in these parameters. The glycemia decreased and higher final body weight increased in DM-RSV when compared with the DM group. The diabetic rats showed higher serum-free fatty acid, which was normalized with RSV. Oxygen consumption (VO_2_) and carbon dioxide production (VCO_2_) decreased (p<0.05) in the DM group. This was accompanied by reductions in RQ. The C-RSV group showed higher VO_2_ and VCO_2_ values. Pyruvate dehydrogenase activity was lower in the DM group and normalizes with RSV. The DM group exhibited higher myocardial β-hydroxyacyl coenzyme-A dehydrogenase and citrate synthase activity, and RSV decreased the activity of these enzymes. The DM group had higher cardiac lactate dehydrogenase compared to the DM-RSV group. Myocardial protein carbonyl was increased in the DM group. RSV increased reduced glutathione in the cardiac tissue of diabetic animals. The glutathione reductase activity was higher in the DM-RSV group compared to the DM group. In conclusion, diabetes is accompanied by cardiac energy metabolism dysfunction and change in the biomarkers of oxidative stress. The cardioprotective effect may be mediated through RVS's ability to normalize free fatty acid oxidation, enhance utilization glucose, and control the biomarkers' level of oxidative stress under diabetic conditions.

## Introduction

Many studies have demonstrated that development of cardiovascular disease is frequently observed in diabetic patients, and it is currently one the major causes that elevates the incidence of morbidity in these patients [1], [2].

The administration of streptozotocin (STZ) results in animal models of the induction of experimental type 1 diabetes mellitus (DM), which is characterized by metabolic disorders and deregulation of energy metabolism, resulting in severe hyperglycemia [3].

The pathogenesis of DM and its complications are associated with the overproduction of reactive oxygen species (ROS) and depletion of the endogenous antioxidant system (enzymatic and non-enzymatic), leading to oxidative stress [4].

Studies have suggested several mechanisms that explain why oxidative stress increases in the diabetic state and may contribute to the cardiac dysfunction. Hyperglycemia exerts an important role on many biochemical pathways that lead to oxidative stress, such as activation of sorbitol (polyol), glucose autoxidation, and non-enzymatic protein glycation [5].

Moreover, alterations in the energy metabolism may also contribute to increased oxidative stress under diabetic conditions. The catabolic processes of nutrients, such as glucose and fatty acids, generate reduced coenzymes (NADH and FAD), which transfer their electrons to the respiratory chain [6], allowing the production of ATP through oxidative phosphorylation. The relative contributions of each of these nutrients as energy sources, depend by their availability in the cell.

The cardiac energy metabolism changes associated with diabetes mellitus contributes to abnormal cardiac function that characterizes heart failure, a condition called diabetic cardiomyopathy [2], [7].

The predominant alteration that occurs in the cardiac metabolism is the suppression of glucose uptake/utilization and the excessive oxidation of fatty acids [2]. This shift in metabolic fuel in the myocardium may be an important factor to the development of diabetic cardiomyopathy [8].

Lipolysis is a metabolic process that occurs in type 1 diabetes mellitus and involves the hydrolysis of triacylglycerols stored in adipose tissue to glycerol and non-esterified fatty acids. As a result, fatty acids are released into the bloodstream, elevating the fatty acid supply to the heart and consequently resulting in excessive oxidation. It was accompanied impairs in myocardial glucose utilization [9].

Since mitochondrial reactive oxygen species production occurs during electron transport to molecular oxygen in the respiratory chain, an influx of electrons, due the excessive fatty acid oxidation, contributes to the generation of additional ROS in the myocardium [10]. Excessive ROS can be the main cause of the pathogenesis of diabetic cardiomyopathy that is associated with increasing circulating fatty acids levels and high β-oxidation in diabetic patients [11].

According to experimental data, the excess of electrons can accumulate in the respiratory chain and raise leakage escape from the respiratory complexes in the inner membrane and partially reduce the molecular oxygen to generate anion superoxide [12], [13]. Thus, other ROS, such hydrogen peroxide and hydroxyl radicals, are formed from superoxide radicals [14]. In energetic terms, excessive myocardial β-oxidation and reciprocal decrease in glucose oxidation reduces the number of molecules of ATP produced per atom of oxygen and decreases cardiac function in type 1 diabetes mellitus [7], [15].

Since ATP from glycolysis is used preferentially by ion carriers' enzymes, inhibition of cardiac glycolysis impairs the intracellular transit of Ca^+2^ in the sarcoplasmic reticulum and consequently lowers cardiac contractility [16].

Hence, strategies that normalize glucose homeostasis or reduce myocardial fatty acid oxidation rates and favor the use of glucose as an oxidative fuel seem to be a therapeutic intervention that improves the energetic metabolism and reduces oxidative stress in the myocardium under diabetic conditions. The utilization of antidiabetogenic and antioxidant agents is becoming increasingly recommended.

Resveratrol (RSV; polyphenol, trans-3,5,4′-trihydroxystilbene) is found in the skin of red grapes, grape juice, and red wines and has a variety of pharmacological properties [17]. RSV has been reported to be efficient antioxidant by its ability to reduce the generation of ROS through directly scavenging oxygen radicals and increasing enzymatic antioxidant defenses in cells [18], [19], specially MnSOD activity in the myocardium [20], and inhibition the oxidation of the low-density lipoprotein [21]. Several studies have shown that the cardioprotective and antidiabetic effects of RSV are mediated by the attenuation of oxidative stress [22], [23], [24].

There is an inverse relationship between the incidence of cardiovascular disease and the consumption of moderate amounts of red wine, a rich source of RSV [25] [26].

Indeed, RSV consumption has been shown to exert beneficial metabolic effects, regulate glucose homeostasis, and protect against chronic metabolic diseases, including diabetes. The RSV can exert the effect cardioprotetor by mechanisms involving modulation and/or activation of myriad targets. RSV active the metabolic sensors SIRT1 (Silent information regulator) and AMPK (AMP-activated protein kinase) [27], [28], which induced the expression eNOS (endothelial NO sythase) [29]. During myocardial infarction and under hypercolesterolemic condition the resveratrol promoted the angiogenesis and reduced of the endothelial dysfunction, respectively, by increasing the expression of the eNOS [29].

The RSV stimulates GLUT 4 (glucose transporter) translocation to the plasma membrane by signaling pathways involving the estrogen receptor [30] and eNOS phophorylated by AMPK [29] allowing the glucose uptake. Other mechanisms include protective action on pancreatic β-cells, probably by expression of SIRT [31].

Palsamy and Subbramanian (2008) reported a lower level of glycosylated hemoglobin in the presence of RSV, showing improved glucose metabolism and preventing the effects of oxidative damage caused by the glycation reaction and consequently attenuating diabetic complications [23].

Thus, the aim of the present study was to investigate the effects of RSV on calorimetric parameters, biomarkers of oxidative stress, and energy metabolism in cardiac tissue of STZ-diabetic rats.

We tested the hypothesis that RSV, through its antihyperglycemic and antioxidant effect, may normalize the myocardial energy metabolism and decrease the biomarkers of the oxidative stress under diabetic conditions.

## Materials and Methods

### Animals and experimental group

Thirty-two male Wistar rats, 60 days of age, were maintained in an environmentally controlled room (22±3°C; 12-hour light/dark cycle and relative humidity of 60±5%) and were fed with a standard rat pellet diet (Purina Ltd., Campinas, SP, Brazil) and water *ad libitum*. The experimental protocol was approved by the Ethics Committee on the Use of Animals (CEUA) at the Institute of Biological Sciences, University of São Paulo State (UNESP), under number 420. The animals were divided into four groups (*n* = 8): C (control group): normal rats; C-RSV: normal rats receiving RSV; DM: diabetic rats; and DM-RSV: diabetics rats receiving RSV. Diabetes mellitus was induced with administration of streptozotocin (STZ; 60 mg/body weight, single dose, i.p.). Blood glucose measured 48 hours after STZ administration and the animals with glycemic levels greater that 250 mg/dL and were considered diabetic. The animals received RSV (1.0 mg/kg/day) for gavage for 30 days. This dose was selected according to a previous studies conducted by Rocha et al. (2009), Huang et al. (2010) and Chang et al. (2011) [21], [32], [33]. Food and water intake were measured daily and body weights were determined once a week.

### Indirect calorimetry

After the experimental period (30 days), the animals fasted overnight (12–14 h) and were placed in metabolic chambers (air flow = 1.0 L/min) coupled to the indirect calorimeter (CWE, Inc, St Paul, USA) to determine the calorimetric parameters: oxygen consumption (VO_2_), carbon dioxide production (VCO_2_), and respiratory quotient (RQ). These measurements were obtained through a respiratory-based software program (software MMX, CWE, Inc., USA) [34].

### Nutritional determination

The energy intake was calculated according Novelli et al. (2007) [35]: -Energy intake (kcal/day)  =  mean food consumption x dietary metabolizable energy (3.81 kcal/g).

### Biochemical parameters in serum and cardiac tissue

After measuring the calorimetric parameters, the animals were anesthetized with a solution (2∶1) of ketamine chloride (10%, Cetamin Syntec Brazil, Ltd.) and xylazine chloride (2%, Xilazin Syntec Brazil, Ltd.) and euthanized by decapitation. The blood was collected and serum separated in a centrifuge at 1,400 *g* for 10 min. Serum was used to determine glycemia by the enzymatic method using glucose oxidase, peroxidase (test Kit Diagnostic LABTEST, Minas Gerais, Brazil), and free fatty acids.

After extraction of the lipid fraction in media containing chloroform, heptane, and methanol, the free fatty acids (FFA) were determined in a reactive mixture containing copper nitrate, triethanolamine, and ammonium hydroxide. After centrifuging (3.000 rpm/5 min), the supernatant is mixed with diethyldithiocarbamate and butanol. Palmitic acid, dissolved in butanol, was used as the standard solution [36].

The heart was removed, and 200-mg samples of the left ventricle were homogenized in sodium phosphate buffer (0.1 M, pH 7.4) using a motor-driven Teflon *Potter Elvehjem*. The samples were then centrifuged at 10,000×g, for 15 min. The supernatant was used for determination of total protein, key enzymes of the energy metabolism and biomarkers of oxidative stress.

The activity of lactate dehydrogenase (LDH) was determined in media containing 50 nM Tris-HCl buffer pH 7.5, 0.15 mM nicotinamide adenine dinucleotide (reduced) (NADH), and 1 mM pyruvate [37]. The activity of pyruvate dehydrogenase (PDH) was assayed using 50 mM potassium phosphate buffer pH 7.4 containing nicotinamide adenine dinucleotide (NAD), thiamine pyrophosphate, coenzyme A, dithiothreitol, MgCl_2_, NBT (nitrobluetetrazoilic), sodium pyruvate, and phenazine methosulfate, and we measured the conversion of pyruvate to acetyl-CoA through the reduction of NAD. Citrate synthase (CS) activity was measured in the presence of Tris-HCl buffer (50 mM, pH 8.0) and several substrates – 0.3 mM acetyl CoA, 0.5 mM oxaloacetate, and 0.1 mM 5,5′-dithiobis-(2-nitrobenzoic) acid (DTNB). The activity of β-hydroxyacyl coenzyme A dehydrogenase (OHADH) was determined in the presence of 50 mM Tris-HCl buffer (pH 7.0), 5 mM EDTA, 0.1 mM acetoacetyl coenzyme A, and 0.5 mM NADH [38].

Lipid hydroperoxide (LH) was measured through hydroperoxide-mediated oxidation of Fe^2+^, with 100 µL of sample and 900 µL of a reaction mixture containing 250 µM FeSO_4_, 25 mM H_2_SO_4_, 100 µM xylenol orange, and 4 mM butylated hydroxytoluene (BHT) in 90% (v/v) methanol [39]. Reduced glutathione (GSH) and glutathione reductase (GR) were measured with a kinetic assay in reaction media containing 2 mM 5,5′-dithiobis-(2-nitrobenzoic) acid (DTNB), 0.2 mM NADPH, and 2 U of glutathione reductase in phosphate buffer (100 mM, pH 7.4) with 5 mM EDTA [40]. The total glutathione (total GSH) was assayed with 0.6 mM DTNB, and 1 U of glutathione reductase in 0.1 M Tris-HCl buffer, pH 8.0 containing 0.5 mM EDTA [40]. Protein carbonyl (PC) was determined according to the method of Reznick and Packer (1994) [41]. Cardiac samples were centrifuged (10,000×g for 10 min) in the presence of 10 mM dinitrophenylhydrazine (DNPH) and 50% trichloroacetic acid (w/v). The pellet was washed three times with ethanol-ethyl acetate (1∶1; v/v) mixture and resuspended in 6 M guanidine hydrochloride. The concentration of carbonyl was quantified at 360 nm using an extinction coefficient of 22 000 M^−1^.cm^−1^.

Enzyme activities were performed using an ELISA reader (Bio-Tech Instruments, Inc., Winooski, USA). The spectrophotometric determinations were performed in an ULTROSPEC Pharmacia Biotech spectrophotometer (Cambridge, England).

### Statistical analysis

The data were evaluated through analysis of variance followed by Tukey's test for comparison between experimental groups. The values are expressed as means ± SD and were considered statistically significant when *p*<0.05 [42].

## Results

Table 1 shows that food, water, and energy consumption were higher in STZ-diabetic rats (DM), while administration of RSV caused marked significantly decreased in these parameters. Diabetic rats had lower final body weight and higher glycemia. The DM-RSV group had significantly decreased glycemia and higher final body weight when compared with the DM group. The untreated diabetic animals exhibited a significant rise in free fat acids. RSV supplementation resulted in the reduction of free fat acid in diabetic rats. Both VO_2_ and VCO_2_, which were detectable in all experimental groups at the end of the experimental period, decreased (p<0.05) for the DM group. This was accompanied by a reduction in RQ. Diabetic rats treated with RSV (DM-RSV) increased in these calorimetric parameters, which were not statistically different from those found in the control group. C-RSV group showed higher values of VO_2_ and VCO_2_ in relation to other groups.

**Table 1:** General characteristics, glycemia, free fatty acids serum level and calorimetric parameters.

Parameters		Groups		
	C	C-RSV	DM	DM-RSV
Food consumption *g/day*	27.82±0.04^b^	26.96±0.14^a^	42.81±0.17^d^	38.30±0.89^c^
Water intake *mL/day*	39.87±2.08^a^	40.05±0.36^a^	206.51±11.67^c^	164.85±14.5^b^
Energy intake *kcal/day*	106.28±0.18^b^	103.00±0.56^a^	163.57±0.65^d^	146.31±3.20^c^
Final body weight *g*	394.36±25.10^c^	381.88±36.56^c^	188.61±33.06^a^	257.09±51.29^b^
Glycemia *mg/dL*	123.09±19.75^a^	132.03±40.30^a^	471.06±73.26^b^	161,40±27,91^a^
FFA *mEq/L*	7.21±0.92^a^	6.11±0,56^a^	9.89±1.07^b^	7.30±0.83^a^
VO_2_ *mL/min*	2.93±0.28^b^	3.43±0.21^c^	1.87±0.15^a^	2.93±0.31^b^
VCO_2_ *mL/min*	2.56±0.27^b^	3.14±0.17^c^	1.47±0.10^a^	2.65±0.28^b^
QR	0.90±0.01^b^	0.91±0.01^b^	0.78±0.04^a^	0.90±0.01^b^

Values are expressed as mean±SD. Control rats (C); resveratrol treated rats (C-RSV); diabetic rats (DM); diabetic rats treated with resveratrol (DM-RSV); free fatty acids (FFA) oxygen consumption (VO_2_); carbon dioxide production (VCO_2_); quotient respiratory (QR).

a, b, c In each row, means followed by different letter indicates statistically significant difference (*p*<0.05).

**Figure 1:**
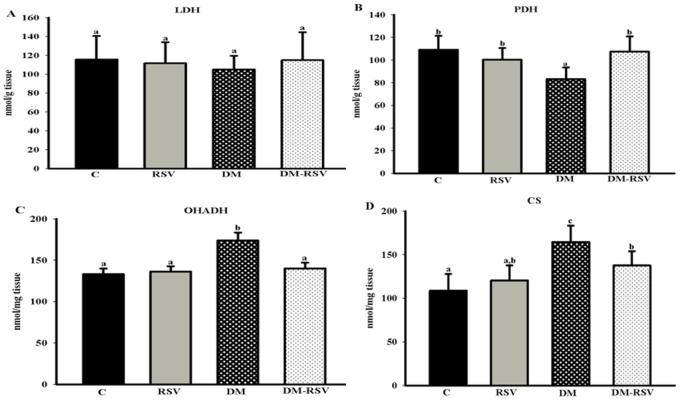
Enzymatic activity in the myocardium. (A) lactate dehydrogenase (LDH); (B) pyruvate dehydrogenase (PHD); (C) β-hydroxyacyl coenzyme-A dehydrogenase (OHADH); (D) citrate synthase (CS) of the different experimental group. Control rats (C); resveratrol treated rats (C-RSV); diabetic rats (DM); diabetic rats treated with resveratrol (DM-RSV); diabetic rats treated with resveratrol (DM-RSV). The results are expressed as the mean±SD. Different superscript letters indicates statistically significant differences with *p*<0.05.

Figure 1 indicates the activity of key enzymes of energy metabolism in the cardiac tissue. No significant differences were found in the activity of LDH. Pyruvate dehydrogenase activity was lower in the DM group and normalizes in presence of RSV. Untreated diabetic animals exhibited higher myocardial OHADH and CS activity. RSV decreased the activity of these enzymes in diabetic rats.

**Figure 2:**
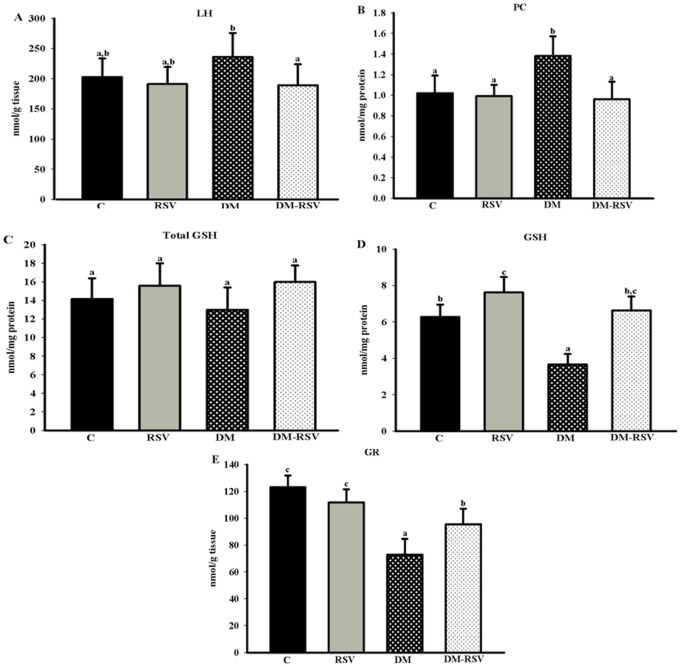
Biomarkers of oxidative stress in the myocardium. (A) lipid hydroperoxide (LH); (B) protein carbonyl (PC); (C) total reduced glutathione (total GSH); (D) reduced glutathione (GSH); (E) glutathione reductase (GR) of the different experimental group. Control rats (C); resveratrol treated rats (C-RSV); diabetic rats (DM); diabetic rats treated with resveratrol (DM-RSV); diabetic rats treated with resveratrol (DM-RSV). The results are expressed as the mean±SD. Different superscript letters indicates statistically significant differences with *p*<0.05.

DM animals had the highest cardiac LH compared with the DM-RSV group, which did not change in the C and C-RSV groups. Myocardial protein carbonyl increased in the DM group in relation to other groups. There were no significant differences in total cardiac GSH. The C-RSV group had higher GSH than the C and DM-RSV groups. RSV increased GSH in the cardiac tissue of diabetic animals (DM-RSV), recovered to values obtained to C group. The glutathione reductase activity was higher in DM-RSV group comparing to DM group. The C and C-RSV groups showed increased glutathione reductase activity when compared to the DM and DM-RSV groups (Figure 2).

## Discussion

There are several mechanisms by which type 1 DM causes disturbances in energy metabolism in cardiac tissue, leading to cardiac dysfunction, such as diabetic cardiomyopathy. A better understanding of the metabolic changes in cardiac tissue in diabetic condition will aid in the development of novel therapies, which could prevent and treat type 1 DM cardiac damage.

Therefore, a model of experimental DM in cardiac muscle is of great interest to clarify its effects on energy metabolism, and it would also be a means for further investigation of the potential use of several effective therapeutic agents to prevent diabetic complications, including diabetic cardiomyopathy.

In the present study, the type 1 DM was induced by administration of STZ, which triggered classic symptoms, such as body weight loss, severe and persistent hyperglycemia, and high food and water intake. It is agreement with other studies [43], [44].

Since STZ causes the destruction of β-cells in pancreatic islets, leading to a deficiency in insulin biosynthesis and secretion, the blood glucose is unavailable as a metabolic fuel for cells from insulin-sensitive tissue (predominantly adipose and muscle tissue), which plays an important role in maintaining glucose homeostasis [45]. Under these conditions, the breakdown of structural protein and lipolysis are increased, contributing to reduce body weight in DM group, despite increased food intake observed in the animals. The administration of RSV in diabetic animals causes reductions in serum glucose and restores the body weight. This could be attributed of improved glycemic control, suggesting greater utilization of glucose by the cell, and consequently lower food, water and energy intake and maintenance of the adipose and muscle tissue in the DM-RSV group.

The literature describes several mechanisms that attempt to explain antihyperglycemic activity of RSV, which stimulate glucose uptake by AMPK [28], [29].

Others authors report that RSV stimulated GLUT4 translocation to the plasma membrane and consequently increases the internalization of glucose in the middle intracellular [30], [46]. The favorable effect of RSV on body weight is consistent with the results of other studies that have demonstrated a restoration of body weight [47], [48] in diabetic rats.

In the present study, we evaluated the metabolic adjustments through indirect calorimetry measurements in treated and untreated diabetic rats. Both O_2_ consumption (VO_2_) and CO_2_ production (VCO_2_) were lower in the DM group compared to the other groups. Although VO_2_ decreased in these animals, the lowest value for the respiratory quotient (RQ = VCO_2_/VO_2_), a marker of substrate utilization, indicates higher lipid oxidation than carbohydrate oxidation. These results are consistent with Wohl et al. (2004), who demonstrated a reduction of the RQ in type 1 diabetes mellitus [49]. Westbrook et al. (2009) reported an inverse relationship between lipid oxidation and RQ, which gives an estimative transition between fat (RQ = 0.7) and carbohydrate (RQ = 1.0) oxidation [50]. Takata et al. (1997) observed an RQ of approximately 0.7 in patients with type 1 diabetes [51]. Under diabetic conditions, the heart has considerable metabolic versatility for fuel selection, e.g., it can decrease uptake and glucose utilization to the detriment of elevated fatty acids oxidation. In addition, the decreased RQ is linked to lower body weight, as observed in the DM group.

The higher VO_2_ and VCO_2_ in DM-RSV group were able to significantly increase the RQ in these animals. Since the RQ varies inversely with lipid oxidation, the higher RQ in the DM-RSV group indicate to decreased fatty acids oxidation and increased carbohydrate oxidation. It can be explain through the ability of RSV to facilitate the entry of glucose into the cell and thus permitting its use as a metabolic fuel.

The serum levels of free fatty acids were enhanced in the DM group (Table 1), this represents an increase in the availability of FFA to the tissues. This finding was related as part to the special metabolic condition in diabetes, where the tissues that are normally insulin-dependent, including cardiac tissues, do not utilize glucose and oxidize fatty acids from lipolysis for energy generation [52].

There is an association between higher circulating free fatty acids, increased myocardial fatty acids uptake/oxidation and reciprocal impaired in glucose oxidation, reflecting a decrease in respiratory quotients [53] with improvements in myocardium contractile performance [54].

The activity of key enzymes can predict which metabolic pathways are being used for energy production [55]. The results of this study indicate altered fuel substrate utilization in cardiac tissue of the DM group. Thus, the preference for fatty acid oxidation can be confirmed by lower PDH (which catalyzes the oxidative decaboxylation of pyruvate from glycolysis) and both increase CS (control of the flux of metabolites through the tricarboxylic acid cycle) and OHADH (biomarker for fatty acid oxidation). This may be demonstrated by the lower RQ in these animals.

Since PHD catalyzes the oxidative decarboxylation of pyruvate derived from glycolysis, the lower PHD activity suppresses glucose oxidation in DM group. In addition, high concentration of FFA in plasma and elevated fatty acid oxidation leads to an increase in acetyl-CoA, which provokes the phosphorylation and inhibition of PDH activity in myocardium [56]. Our results compared with these studies suggest that excessive FFA oxidation exerts a negative impact on glucose oxidation in the cardiac tissue of diabetic animals. However, these metabolic abnormalities in cardiac myocytes, under diabetic conditions, contribute to the pathogenesis of diabetic cardiomyopathy [2].

Treatment with RSV decreases the serum level of FFA, increases PHD activity, and reduces OHADH activity in the myocardium of diabetic animals (RSV-DM). This suggests that the RSV-DM group had increased peripheral glucose utilization as an energy substrate and decreased FFA oxidation, which can be demonstrated by greater RQ in these animals. These findings would be consistent with previous studies reporting the association between the rise in RQ and lower fat oxidation after supplementation with RSV [57]. Furthermore, PHD activity correlates with glucose oxidation in human skeletal muscle [58]. However, these observations indicated that RSV protects the metabolic shifting in cardiac tissue and hence cellular energy homeostasis and maintenance of the glycolytic pathway. Since the substrate for glycolytic enzymes is available to be bound to the sarcoplasmic reticulum, which depends on the ATP generated by glycolysis [16], [59], the supplementation of RSV becomes important in controlling cardiac contractility and improves heart function under diabetic conditions.

Interestingly, LDH, the enzyme responsible for the inter-conversion of pyruvate to lactate, was unchanged in cardiac tissue, indicating the absence of anaerobic glycolysis in diabetic animals.

Evidence suggests that oxidative stress is a key player and contributor to diabetes-stimulated cardiac dysfunction, which may be associated with alterations in energy metabolism, i.e., greater oxidation of fatty acids in relation the oxidation of glucose. The mitochondrial oxidation of fatty acids leads to greater oxygen requirements and consequently increased ROS formation in the electron transport chain due to incompletely reduced of oxygen [53], [9]. Furthermore, electron transfer donors (NADH and FADH), generated during the citric acid cycle and β-oxidation raise the electron flux in the respiratory chain and the rate of ROS generation [60].

ROS is related to the development of oxidative stress, which often occurs in the diabetic state. In fact, the accumulation of the biomarkers of oxidative stress was verified at the end of the experimental period in the DM group. These results are consistent with other studies of serum [52], pancreatic tissue [61], and adipocytes [62].

Since lipid hydroperoxide (LH) and protein carbonyl are important products formed as result of peroxidation of the polyunsaturated fatty acids in biomembranes and oxidative modification of protein mediated by ROS, respectively [52], [63], the decrease in these biomarkers in cardiac tissue of diabetic animals (the DM-RSV group) reflects the ability of RSV as an antioxidant compound. The antioxidant property of RSV is well documented, and several studies have demonstrated that it supplementation declined the lipid peroxidation [64].

GSH is an important component in the non-enzymatic antioxidant defense system, and when the depletion of GSH exceeds the capacity of regeneration of this molecule, an alteration occurs in the intracellular GSH homeostasis that contributes to oxidative stress. The GSH acts as cofactor of glutathione peroxidase (converts H_2_O_2_ to H_2_O) and becomes oxidized GSSG, which returns to the reduced form (GSH) through a reaction catalyzed by GR. In the present study, diabetic rats had lower cardiac GSH concentration and decrease GR activity. The decreased GSH levels in red blood cells from diabetic patients was attributed possibly by reducing of the pentose phosphate pathway [65]. This fact could explain the results obtained, since the activity of GR depends on the intracellular concentration of NADPH, which becomes oxidized (NADP^+^) and converted to its reduced form in the pentose phosphate pathway.

Alterations in intracellular redox status involving the glutathione system under diabetic conditions have been reported by other studies, such as the decreased GSH level in hepatic, tissue [66] and in aortic tissue [67].

The present study showed that RSV increased the cardiac content of GSH in the DM-RSV group, probably in response to the major activity of GR in the myocardium of these animals. The decrease in the rate of oxidation of glucose has been associated with declining intracellular concentrations of NADPH [4]. Thus, treatment with RSV in diabetic rats increased GR activity, probably due to improvement in glucose oxidation, which allowed the reduction of NADP^+^ by the pentose phosphate pathway.

Furthermore, the ability of RSV to raise GSH is consistent with Kode et al. (2008), who observed increased GSH synthesis in the presence of RSV [68]. Shankar et al., (2007) reported that RSV may act directly by scavenging the ROS or increase the endogenous antioxidant defense [19], corroborating the results obtained in this study, because RSV decreased the concentration of biomarkers of oxidative stress produced in diabetes mellitus, possibly by maintaining GSH status in the cardiac tissue of rats belong to the DM-RSV group.

In conclusion, the diabetic state induces a shift in the metabolic pathway for energy production, worsening glycemia and raising LH and PC, important toxic intermediates in the development of cardiac oxidative stress. RSV recovered glucose homeostasis, normalized free fatty acid oxidation, enhanced utilization glucose, regulated myocardial metabolic enzymes and calorimetric parameters, and optimized cardiac energy metabolism in diabetes conditions. The beneficial effect of RSV on glycemia and energy metabolism may contribute to normalizing the level of biomarkers of oxidative stress. These findings provide information that can guide future studies aimed at finding therapeutic alternatives for diabetic complications, especially those related to cardiomyopathies.
